# Promotion of Cancer Cell Invasiveness and Metastasis Emergence Caused by Olfactory Receptor Stimulation

**DOI:** 10.1371/journal.pone.0085110

**Published:** 2014-01-08

**Authors:** Guenhaël Sanz, Isabelle Leray, Aurélie Dewaele, Julien Sobilo, Stéphanie Lerondel, Stéphan Bouet, Denise Grébert, Régine Monnerie, Edith Pajot-Augy, Lluis M. Mir

**Affiliations:** 1 INRA, UR1197 Neurobiologie de l'Olfaction et Modélisation en Imagerie, Jouy-en-Josas, France; 2 IFR 144 NeuroSud Paris, Gif-sur-Yvette, France; 3 CNRS, UMR8203 Vectorologie et thérapeutiques anti-cancéreuses, Institut Gustave-Roussy, Villejuif, France; 4 Univ. Paris-Sud, UMR8203, Orsay, France; 5 CNRS-TAAM, UPS44, Centre d’Imagerie du Petit Animal, Orléans, France; 6 INRA, UMR 1313, Génétique Animale et Biologie Intégrative, Jouy-en-Josas, France; 7 CEA, DSV, IRCM, SREIT, Laboratoire de Radiobiologie et Etude du Génome, Jouy-en-Josas, France; 8 AgroParisTech, UMR 1313, Génétique Animale et Biologie Intégrative, Jouy-en-Josas, France; Wayne State University School of Medicine, United States of America

## Abstract

Olfactory receptors (ORs) are expressed in the olfactory epithelium, where they detect odorants, but also in other tissues with additional functions. Some ORs are even overexpressed in tumor cells. In this study, we identified ORs expressed in enterochromaffin tumor cells by RT-PCR, showing that single cells can co-express several ORs. Some of the receptors identified were already reported in other tumors, but they are orphan (without known ligand), as it is the case for most of the hundreds of human ORs. Thus, genes coding for human ORs with known ligands were transfected into these cells, expressing functional heterologous ORs. The *in vitro* stimulation of these cells by the corresponding OR odorant agonists promoted cell invasion of collagen gels. Using LNCaP prostate cancer cells, the stimulation of the PSGR (Prostate Specific G protein-coupled Receptor), an endogenously overexpressed OR, by β-ionone, its odorant agonist, resulted in the same phenotypic change. We also showed the involvement of a PI3 kinase γ dependent signaling pathway in this promotion of tumor cell invasiveness triggered by OR stimulation. Finally, after subcutaneous inoculation of LNCaP cells into NSG immunodeficient mice, the *in vivo* stimulation of these cells by the PSGR agonist β-ionone significantly enhanced metastasis emergence and spreading.

## Introduction

Olfactory receptors (ORs) are G protein-coupled receptors mainly expressed in olfactory sensory neurons (OSNs) of the olfactory epithelium, where they detect and discriminate myriads of odorants according to a combinatorial code in which an OR can be activated by various odorants and an odorant can stimulate various ORs [1], [2]. Moreover, ORs are expressed in non-olfactory tissues [3]–[5] where they can play additional roles. They notably govern sperm chemotaxis, regulate migration and adhesion of muscle cells, and control serotonin secretion by enterochromaffin (EC) cells [6]–[10]. Several studies also reported that some ORs can be tumor marker, one of them modifying *in vitro* the proliferation of LNCaP prostate cancer cells [11], [12], [13], [14].

In particular, EC cells can acquire a tumoral phenotype and differentially express ORs depending on the neuroendocrine carcinoma evolution [15]. The BON cells, a human EC cell line derived from a metastasis of a pancreatic carcinoma [16], [17], were described to endogenously express ORs [8] which could be tumor markers when overexpressed [15]. Because BON cells were derived from a metastasis, we explored whether activation of ORs by agonist odorants could have a role in tumor progression. To this end, we decided to identify the ORs expressed in BON cells. However the agonist or antagonist odorants specific of BON cells ORs are unknown, like for most of the hundreds of identified human ORs. We thus tried to develop a model by transfecting these cells with deorphanized ORs. The heterologous expression achieved allowed us to assess the *in vitro* invasiveness of these cells upon stimulation with the odorant ligand of the transfected receptor. Furthermore, we identified PI3 kinase γPI3Kγ as a component of the signaling pathway induced by OR stimulation and promoting cell invasiveness. A more physiological model was also used *in vitro*, the LNCaP prostate cancer cells which overexpress the PSGR (Prostate Specific G protein-coupled Receptor), an endogenous and deorphanized OR considered as a tumor marker, and wich was described to inhibit the proliferation of these cells *in vitro* [12]. This model was then used *in vivo* to analyze the role of ORs stimulation in tumor progression, that is in metastasis emergence and spreading.

## Materials and Methods

### Ethics Statement

The animals were handled in conformity with the Guidelines of the French government regarding operative procedures and animal care. Protocol was approved by the ethics comity for experiments with animals named “Comité d’Ethique en Expérimentation Animale de l’IRCIV” CEEA-26 (protocol number 2012-043).

### Reverse transcription (RT)-PCR, cloning and sequencing

Total RNAs were extracted using TRIzol reagent (Invitrogen) and treated with DNase I. RT was performed with the « SuperScript First-Strand®, Synthesis System for RT-PCR» kit (Invitrogen).

For single cell RT-PCR, single cells were collected by aspiration into a glass pipette and RT was performed using the « Single Cell Superscript™ III Cells Direct cDNA Synthesis System » kit (Invitrogen) after cell disruption, protein denaturation and DNAse treatment.

Nested PCR was carried out starting from 1 µL of RT products and using degenerate primers targeting OR conserved regions, or primers specifically targeting OR identified with the degenerate primers. Degenerate primers sequences were kindly provided by Stephan Bieri (Givaudan, Switzerland). Absence of genomic DNA was controlled using human GAPDH or β-actin primers on DNase I-treated RNAs without reverse transcriptase.

PCR products amplified with degenerate primers were cloned into the pGEM-T vector (Promega) and sequenced by Beckman Coulter Genomics. PCR products amplified with specific primers were directly sequenced (Beckman Coulter Genomics).

### Chemicals

Odorants, DMSO and mineral oil (M3516) were purchased from Sigma-Aldrich, Fluka or Acros Organics at the highest purity available. AS605240 was purchased from Euromedex (Selleck, S1410) and gallein from TOCRIS bioscience. Paraffin (CellWax) was obtained from CML, and hemalun, eosin, and safran from RAL.

### Mammalian expression vectors

OR1G1 or OR17-40 coding sequences were introduced into the pCMV-Tag3B mammalian expression vector (Stratagene) in a way resulting in the fusion of a cmyc epitope at the receptor N-terminus. The resulting vectors were named pCMV-TagOR1G1 and pCMV-TagOR17-40.

### Cell culture and transfection

BON cells (subclone #7) were kindly provided by Dr Courtney M. Townsend (Department of Surgery UTMB, Galveston, TX 77551, USA) [16], [17]. They were grown in DMEM/F-12 (Ham) without phenol red (GIBCO, Invitrogen Corporation) supplemented with 10% fetal bovine serum (Hyclone, Perbio) and antibiotics (100 U penicillin/mL and 100 µg streptomycin/mL, Invitrogen), at 37°C in a humidified incubator with 5% CO_2_. Cells were transiently transfected with pCMV-TagOR1G1 or pCMV-TagOR17-40 using jetPEI™(Polyplus-transfection) according to the manufacturer’s instructions. OR expression at the cell surface was checked by immunofluorescence microscopy using the monoclonal anti-cmyc-Cy3 antibody (C6594, Sigma-Aldrich) on non-permeabilised cells.

LNCaP cells were purchased from ATCC (Clone FGC, No. CRL-1740™) at passage 19, and grown in RPMI 1640 medium (ATCC, No. 30-2001) supplemented with 10% fetal bovine serum (ATCC, No. 30-2021), at 37°C in a humidified incubator with 5% CO_2_.

### Calcium imaging

BON and LNCaP cells were seeded onto a 96-well culture plate (black microtiter plate, Greiner Bio-one), respectively at a density of 10^5^ and 0.5×10^5^ cells per well. 24 hours later, cells were loaded with 2.5 µM of fluo-4 acetoxymethyl ester (Molecular Probes), as previously described [18]. Calcium imaging was performed using an inverted epifluorescence microscope (CK40 Olympus) equipped with a digital camera (ORCA-ER, Hamamatsu Photonics). Ca^2+^ reponses were observed at 460–490 nm excitation and ≥ 515 nm emission wavelengths. Data acquisition and analysis was performed using the SimplePCI software (Hamamatsu, Compix). Odorants and mineral oil were prepared extemporaneously by a first dilution into DMSO and then serial dilutions into Hanks’ salt solution (Eurobio) supplemented with 20 mM Hepes, pH 7.2. Stimuli were tested at concentrations that do not elicit calcium responses in mock-transfected cells. 1 µM isoproterenol (Sigma-Aldrich) was applied as a positive control. The Ca^2+^ signal was measured as the relative change in fluorescence intensity ΔF/F =  (F–F_0_)/F_0_, where F_0_ is the fluorescence level before stimulation. Results were expressed as the mean of the ΔF/F of at least twenty cells.

### *In vitro* assessment of cell invasion

Collagen type I gels were prepared as described by De Wever [19]. Cells were cultured for 48 hours before seeding them as a suspension of single cells deposited on top of the collagen type I gels. To stimulate ORs, odorants and mineral oil were first diluted into DMSO and then into the collagen I solution or the culture medium. The effects of specific inhibitors of PI3Kγ (AS605240) and βγ subunits of G proteins (gallein) were also assessed by adding them to the collagen gel and culture medium. For controls, DMSO was added at the same final concentration used to dilute these chemicals. The amount of added DMSO did not exceed 0.2% and did not modify the number of invasive cells compared to tests without DMSO (data not shown). 24 hours after their seeding, invasive cells presenting invasive extensions into the collagen gel and non-invading cells were counted in 10–15 microscope fields randomly selected. Results were expressed as the percentage of invasive cells (invasion index). Morerover, F-actin cytoskeleton was observed using rhodamine-conjugated phalloidin (Invitrogen). For this purpose, cells were fixed for 20 min with 3% paraformaldehyde in PBS at room temperature, then permeabilized for 15 min with 0.5% Triton X-100 in PBS and blocked for 30 min with 2% BSA, 1% glycine in PBS. Cells were incubated with rhodamine-phalloidin (1∶300) in PBS for 30 min at room temperature and extensively washed with PBS before observation with an inverted epifluorescence microscope.

### Mice

Nod Scid Gamma (NSG) male mice were bred in the animal housing facilities of the Institut Gustave Roussy, with free access to food and water. Plastic cages were connected to controlled ventilated racks. The cages with the animals exposed to the odorant β-ionone were connected to a separated ventilation unit.

### *In vivo* assessment of cell invasion and metastases

LNCaP cells at passage 25 were inoculated into 8 week-old castrated male NSG mice (castration was performed two weeks before cell inoculation). 10^6^ cells were suspended in 75 µL of RPMI 1640 plus 75 µL of Matrigel (BD Biosciences) and injected with a needle (26G) into the subcutaneous space, at 2 sites in each flank of the mice. The odorant β-ionone was first diluted into DMSO at a concentration of 100 mM and then into the RPMI + Matrigel mixture at the final concentration of 100 µM. DMSO was also added at the same dose to the RPMI + Matrigel mixture without odorant. A first group of 5 mice was inoculated with LNCaP cells (in the presence of DMSO) and received no further treatment. Five other mice were inoculated with LNCaP cells (in the presence of DMSO) and brushed with mineral oil three times a day during 6 weeks and then three times a week until sacrifice. A third group of 5 mice was inoculated with LNCaP cells in the presence of β-ionone in DMSO. These mice were brushed with 1 mM β-ionone directly diluted in mineral oil three times a day during 6 weeks and then three times a week until sacrifice. Before sacrifice, some animals were first examined by tomoscintigraphy (SPECT, NanoSPECT/CT Bioscan) using ^99m^Tc-MDP, a classical bone scintigraphy agent for functional imaging of the bone. This analysis was not performed on all animals because it appeared less informative than X rays in our study. Thus all mice were explored *in vivo* by microcomputed tomography (µCT) (CT120, General Electric Healthcare) to detect bone metastasis. 360 X ray projections were collected in 1° increments (100 kVp, 50 mA, 20 msec exposure) for about 5 min total scan time. Images were reconstructed into 3D volumes (50 µm resolution) on a reconstruction cluster using a modified tent-FDK conebeam algorithm (GE reconstruction software). 3D data were processed using MicroView (GE Healthcare). Data analysis was performed first on individual slices (axial, coronal, sagittal) then on reconstructed volumes and MIP images (Maximum Intensity Projection). Animals were sacrificed when tumor size exceeded 1,500 mm^3^. Upon autopsy, tumors and tissues known to harbor metastases from prostate tumors such as lymph nodes, lungs and spines, were sampled. Livers and Tyson glands were also sampled, some livers appearing anomalous and some Tyson glands surprisingly large. Tissues were fixed for 24 hours in formaldehyde then stored in 70% ethanol at 4°C. For spines, decalcification was realized by an additional incubation in 10% EDTA, pH 7.4, at 4°C during one week. All samples were dehydrated in ethanol and included in paraffin. Serial sections of 5 µm thickness were prepared and dewaxed in toluene and rehydrated in ethanol and then water. Some sections were stained with hemalun (RAL), eosin and safran (HES staining). Immunohistochemistry was performed on other sections using anti-PSGR (LS-A6332, Cliniscience), anti-PSA (ab9537, abcam), or rabbit serum as a negative control, the Vectastain Elite ABC-Peroxidase Kits Rabbit IgG (Cliniscience), and a DAB revelation (SK-4100, Vector).

## Results

### ORs endogenously expressed by BON cells

Since BON cells display an heterogeneous morphology, we isolated homogeneous subclones. OR expression was investigated by nested PCR on cDNAs from nine clones using degenerate primers targeting OR conserved regions, and PCR products sequencing. We detected ORs transcripts in six of the clones (Table S1). Among them, five displayed expression of more than one OR gene or pseudogene, and the panel of ORs identified varied from clone to clone. To confirm these results, we performed nested PCR with primers specifically targeting the previously identified ORs. Actually all nine clones expressed ORs transcripts (Table 1) and some of them (OR7D2, OR1F1) were found in most of the clones. It must be highlighted that OR7A17, OR7D2 and OR2A1 transcripts are also found in several tumors (ESTs listed in the HORDE database).

**Table 1:** Targeted search of ORs expressed by subclones of BON cells, using nested RT-PCR with primers specific to previously identified ORs.

Clone	OR4F16	OR13H1	OR7A17	OR10Q1	OR7D2	OR2A1	OR6V1	OR1F1	OR13A1
1	X			X	X		X	X	
3	X				X			X	
4	X	X			X				
8	X			X	X			X	
9				X	X			X	
10		X		X	X			X	
14	X	X	X	X	X			X	
19	X				X			X	X
23	X	X				X	X	X	X

(X) indicates identified ORs.

To further assess that, contrary to OSNs, BON cells co-express several ORs, we analyzed OR expression at the single-cell level. We succeeded in amplifying cDNAs corresponding to GAPDH or β-actin for most tested cells, but OR cDNAs could be amplified only for a few cells, probably because of the very low level of OR mRNAs at the single-cell level. Our data show that some single BON cells do co-express more than one OR transcript (Figure S1a).

### Heterologous functional expression of ORs in BON cells

**Figure 1:**
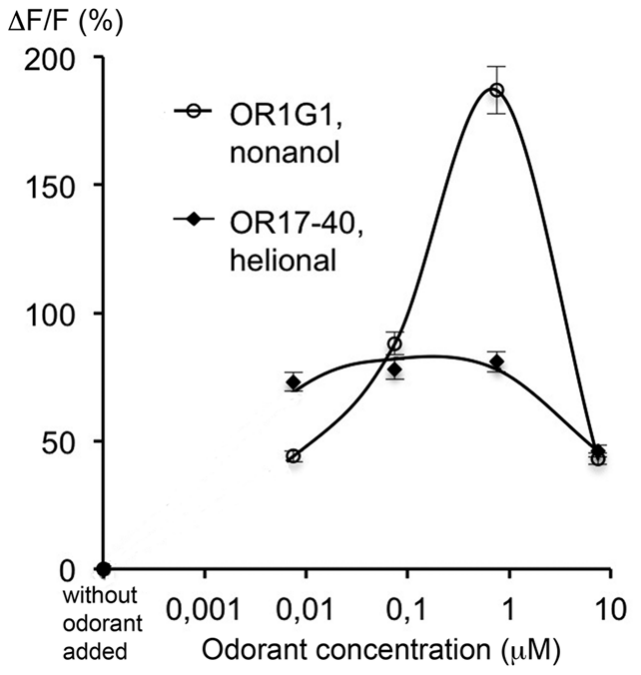
Functional response of ORs heterologously expressed in BON cells. BON cells were transiently transfected to express OR1G1 or OR17-40 receptors. 72h later, cells were loaded with fluo-4 and stimulated with the respective odorant ligands of the transfected ORs (1-nonanol and helional). Calcium responses due to the interaction between the OR and its specific odorant agonist are expressed as the mean fluorescence variation ΔF/F (%). (open circles) OR1G1 cells, 1-nonanol ; (filled diamonds) OR17-40 cells, helional ; bars indicate standard deviation (n = 3). Mock-transfected cells did not respond to 1-nonanol nor helional.

Since BON cells endogenously express ORs, we infered that they could also heterologously express functional ORs after transfection of the OR1G1 and OR17-40 genes. BON cells appeared to express these heterologous receptors and to expose them at the plasma membrane (Figure S1b). We also found in BON cells the transcript of REEP1, a protein which facilitates OR expression in OSNs [20] (Figure S1c). We then demonstrated that the heterologously expressed receptors are functional, inducing a calcium response when they are stimulated with their respective ligand (1-nonanol for OR1G1 and helional for OR17-40 [18], [21]) (Figure 1). The calcium response induced by stimulation of the OR17-40 receptor is less pronounced than that induced by stimulation of the OR1G1 receptor, but it remains significant. Differences between OR response levels can be due to different expression levels of the receptors, to a different coupling efficiency with the endogenous G-proteins of heterologous cells, or to a different efficiency of the ligands used. Mock-transfected cells did not respond to nonanol nor helional, showing that the odorants tested are not agonists of the ORs endogenously expressed in BON cells.

### OR-induced enhancement of cell invasiveness

**Figure 2:**
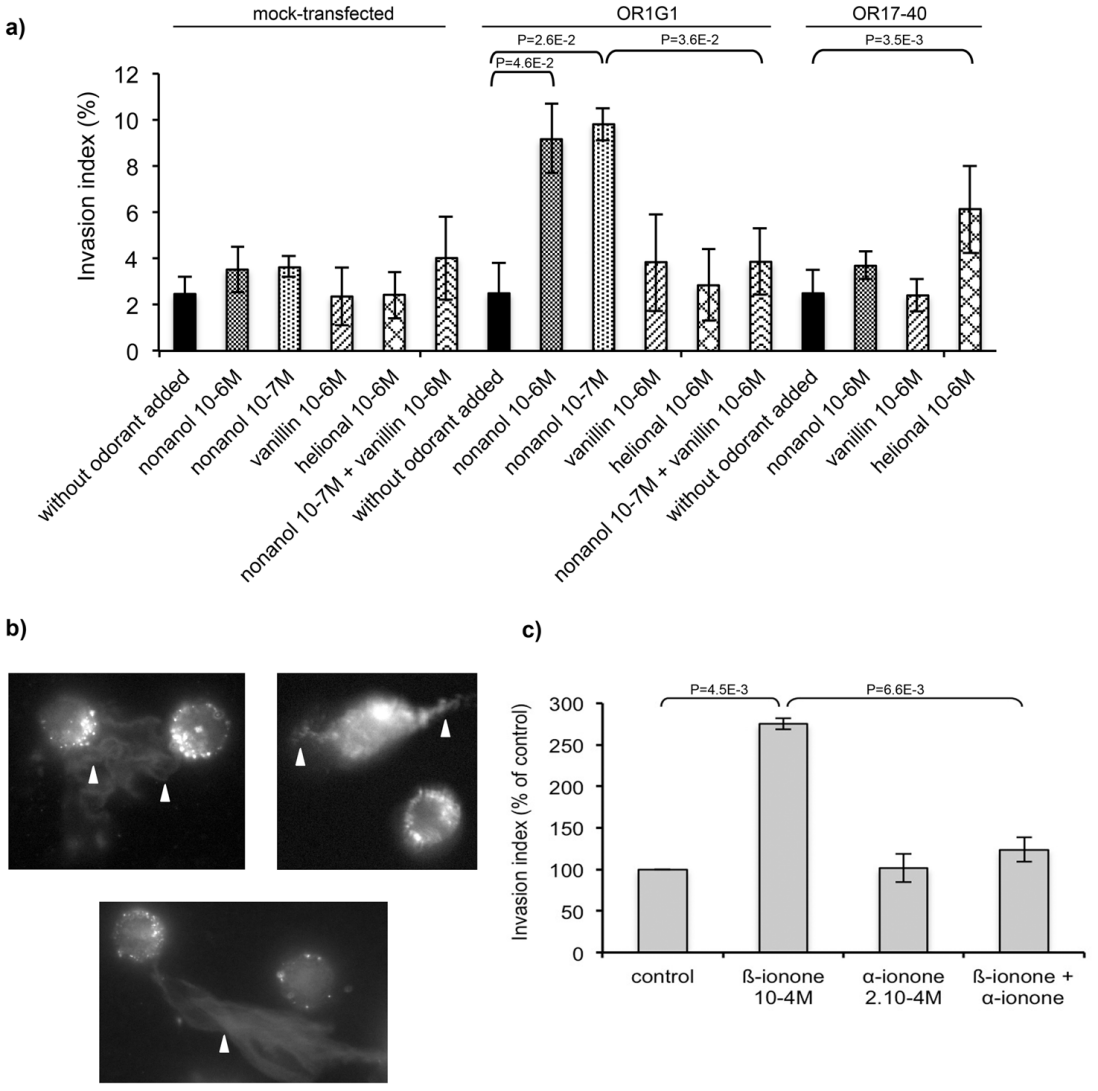
Promotion of cancer cells invasiveness upon odorant stimulation. (**a**) BON cells were transiently transfected to express OR1G1 or OR17-40 receptors or mock-transfected. Cells were seeded on collagen type I gels and stimulated by the respective odorant ligands of OR1G1 and OR17-40 receptors (nonanol: OR1G1 agonist, vanillin: OR1G1 antagonist, helional: OR17-40 agonist). Invasive cells were counted 24 hours later. Results are presented as the invasion index. (**b**) Modification of the F-actin cytoskeleton of BON cells in collagen type I matrices. F-actin was revealed by rhodamine-conjugated phalloidin. Invasive extensions into collagen gels characterizing invasive cells are indicated by arrows. (**c**) LNCaP cells were seeded onto collagen type I gels and stimulated by PSGR ligands (β-ionone: agonist, α-ionone: antagonist). Invasive cells were counted 24 hours later. Results are presented as the invasion index relative to control cells without odorant stimulation. Standard deviation of the control was 13,42%. Statistics were performed using a two-tailed Student test and bars indicate standard deviation (n = 3).

Using BON cells heterologously expressing OR1G1 or OR17-40 receptors, we assessed the invasiveness of collagen type I gels [19] by these cells, stimulated or not with the odorant agonists of these ORs. In absence of odorant stimulation, the invasiveness of BON cells was not modified by heterologous expression of ORs (the invasion index remains around 3%, Figure 2a). Nonanol stimulation increased significantly the invasion index of OR1G1-expressing cells (OR1G1 cells) by a factor of 2.7, whereas helional stimulation increased the invasion index of OR17-40 cells by a factor of 2.5 (Figure 2a). We observed that 10^−6^ and 10^−7^ M of nonanol induced the same invasion level, whereas 10^−6^ M appeared more efficient in activating OR1G1 in calcium imaging experiments. This may be due to the inability of BON cells to reach larger invasion levels (around 10% invasive cells). Nonanol and helional had no significant impact on mock-transfected control cells. Nonanol had no significant effect on OR17-40 expressing cells, nor helional on OR1G1 expressing cells. Furthermore, vanillin, an antagonist of the OR1G1 receptor [22], was able to specifically counteract the invasiveness induced by nonanol in OR1G1 cells. The invasion index of control cells stimulated by nonanol alone or by a mixture of nonanol and vanillin was unchanged (Figure 2a). Invasive cellular extensions into collagen type I gels, characterizing the invasive cells, were also observed after immunolabeling of the F-actin cystoskeleton (Figure 2b). All together, these results demonstrate that, *in vitro,* ORs stimulation by odorants can specifically promote invasiveness of the OR-expressing cancer cells.

We confirmed this result using the LNCaP prostate cancer cells which endogenously express an OR, the PSGR. This receptor has known agonist and antagonist odorants [12], respectively the β-ionone and α-ionone. We used a 100 µM concentration of β-ionone to stimulate LNCaP cells, since this dose was already reported to stimulate the PSGR [12] and it induced the highest invasiveness of LNCaP cells in our hands (data not shown). As shown in Figure 2c, stimulation of PSGR with 100 µM β-ionone increased invasiveness of LNCaP cells by a factor of 2.75 and this effect was totally abrogated by the antagonist α-ionone. Alone, this antagonist had no effect on LNCaP cells invasion level. While there is no negative control with LNCaP cells that would not express PSGR, the drastic pharmacological effect of α-ionone argues in favor of a specific effect of β-ionone through PSGR stimulation. Exclusion of a non specific chemical effect of β-ionone on LNCaP cells inducing invasiveness is also supported by the fact that α-ionone, which is very similar to β-ionone and was applied at twice the β-ionone dose, did not induce invasiveness of LNCaP cells. Moreover, we tested the effect of 100 µM β-ionone on the invasiveness of PC3 cells, other prostate cancer cells that do not express the PSGR [12], and we did not observe an increased invasiveness in these cells. Experimental results detailed below also support the idea that LNCaP invasiveness can be enhanced through PSGR stimulation.

### Involvement of PI3Kγ in cell invasiveness induced by ORs

**Figure 3:**
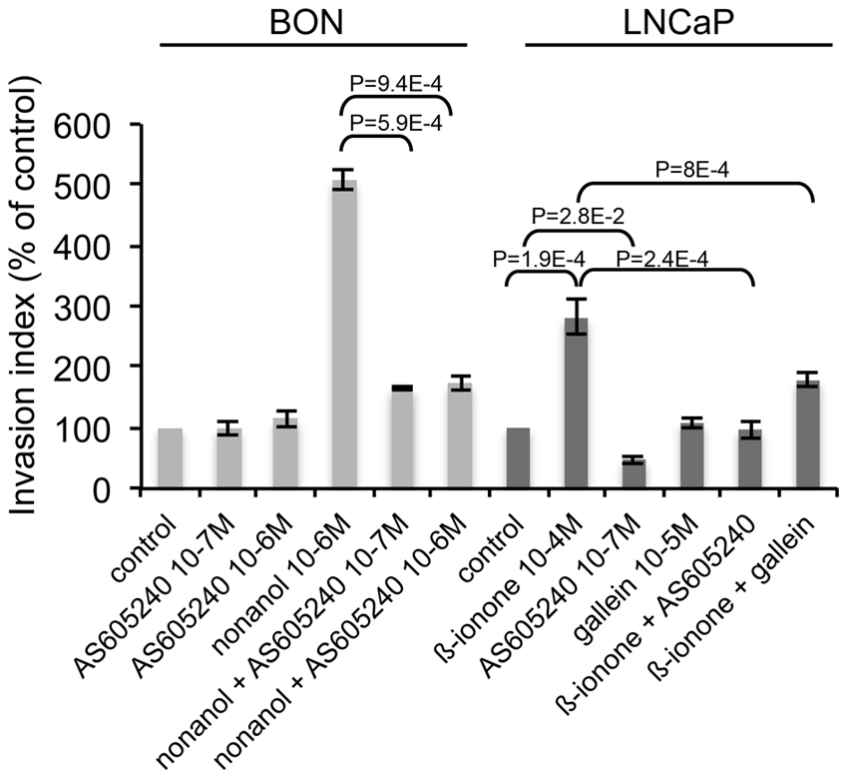
PI3Kγ involvement in cell invasiveness promoted by ORs. BON cells heterologously expressing the OR1G1 receptor were seeded onto collagen type I gels and were stimulated by nonanol (OR1G1 agonist) in the presence of a specific PI3Kγ inhibitor (AS605240). LNCaP cells were seeded onto collagen type I gels and were stimulated by β-ionone (PSGR agonist) in the presence of a specific PI3Kγ inhibitor (AS605240) or an inhibitor of βγ subunits of G proteins (gallein). Invasive cells were counted 24 hours later. Results are presented as the invasion index relative to that of control cells (with no odorant nor AS605240 nor gallein). Standard deviation of the control was 4,74% for the control BON cells and 13,86% for the control LNCaP cells. Statistics were performed using a two-tailed Student test and bars indicate standard deviation (n = 3).

PI3Kγ activation through GPCRs can be involved in transforming functions such as invasion [23], and a crosstalk between odorant signaling and PI3Kγ was described in olfactory sensory neurons [24], [25]. We thus explored whether PI3Kγ could be part of the signaling pathway which is triggered by the odorant activation of ORs and promotes cell invasiveness. First we showed the expression of PI3Kγ in BON and LNCaP cells by crude lysates immunoblotting with an antibody targeting PI3Kγ (data not shown). We then assessed the invasiveness of BON cells hetorologously expressing OR1G1 or of LNCaP cells upon stimulation with agonists of OR1G1 or PSGR, in the presence of a specific inhibitor of PI3Kγ (AS605240). 10^−6^M of AS605240 have been reported to completely inhibit PI3Kγ [26]. Concerning BON cells, using 10^−6^M and 10^−7^M of AS605240, we found a similarly large (about 80%) but not total reduction of the cell invasiveness promoted by OR1G1 upon nonanol stimulation (Figure 3), indicating that the maximal effect is observed at 10^−7^M of AS605240. Thus, PI3Kγ appears to play a major role in mediating BON cell invasiveness promoted by the OR stimulation by its specific odorant, even if other signaling pathways might also be involved. Involvement of PI3Kγ was confirmed for LNCaP cells (Figure 3). However, contrary to BON cells, PI3Kγ inhibitor AS605240 induced a reduction of LNCaP invasiveness even in absence of PSGR stimulation. Therefore PI3Kγ seems to be also involved in the basal invasiveness of LNCaP cells. Moreover, since PI3Kγ can be activated by the G_βγ_ subunit of the G proteins through GPCR activation [27], we used gallein, a G_βγ_ subunits inhibitor that interferes with the interaction of G_βγ_ subunits with PI3Kγ [28], and showed that it counteracted the enhancement of LNCaP cell invasiveness induced by PSGR stimulation (Figure 3). This result also supports the involvement of PI3Kγ in the invasiveness of tumor cells induced by OR stimulation.

### OR activation-induced enhancement of cancer cell invasiveness *in vivo*

**Figure 4:**
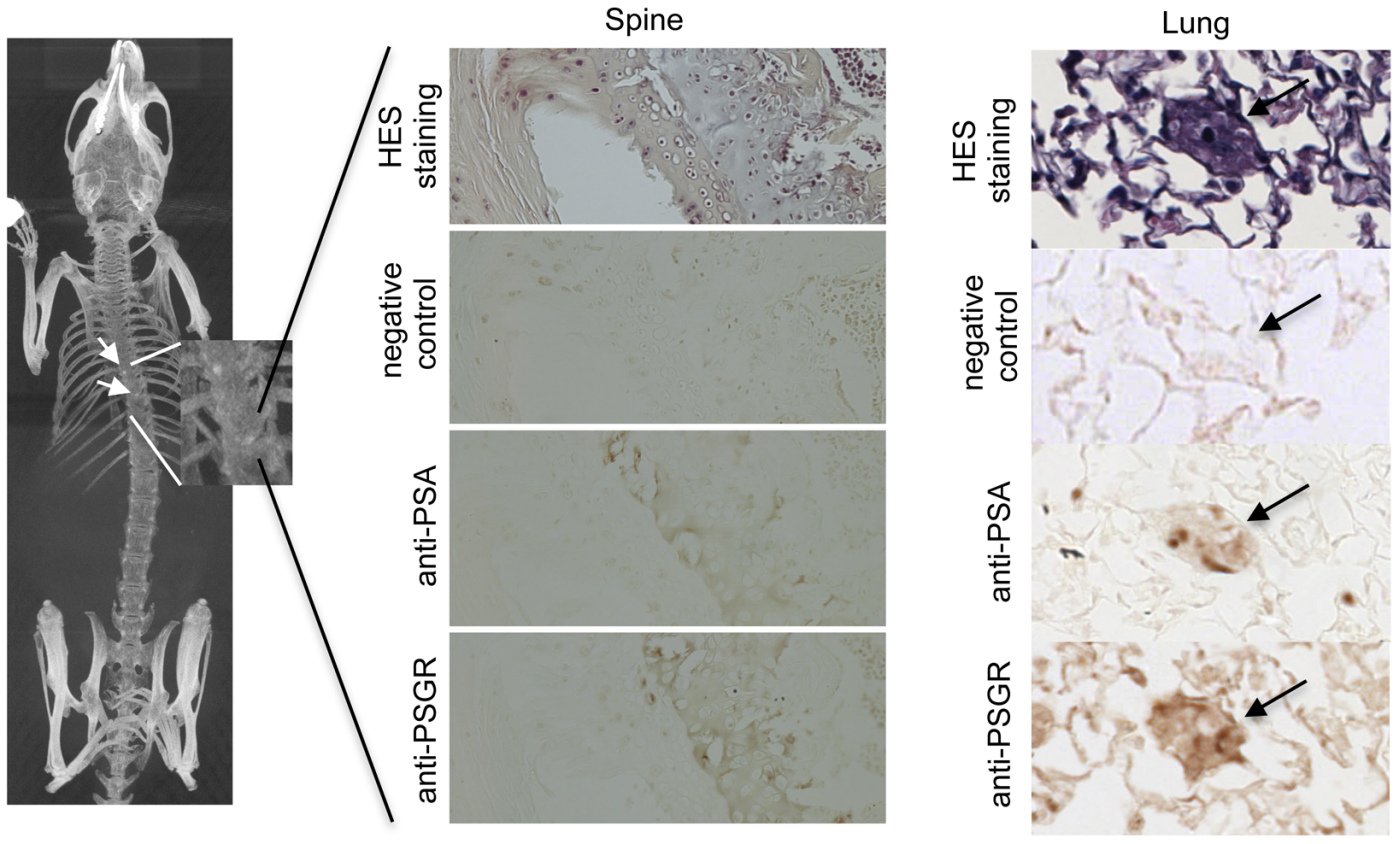
Examples of metastases found in the spine or the lungs of immunodeficient mice inoculated with LNCaP cells. Metastases are indicated by arrows. In the spine, metastases were observed using microcomputed tomography (Maximum Intensity Projection) and confirmed post-mortem by HES staining and immunohistology using anti-PSA (prostate specific antigen) and anti-PSGR antibodies (only one metastasis is presented). In the lungs, metastases were characterized by HES staining and immunohistology using anti-PSA and anti-PSGR antibodies.

**Figure 5:**
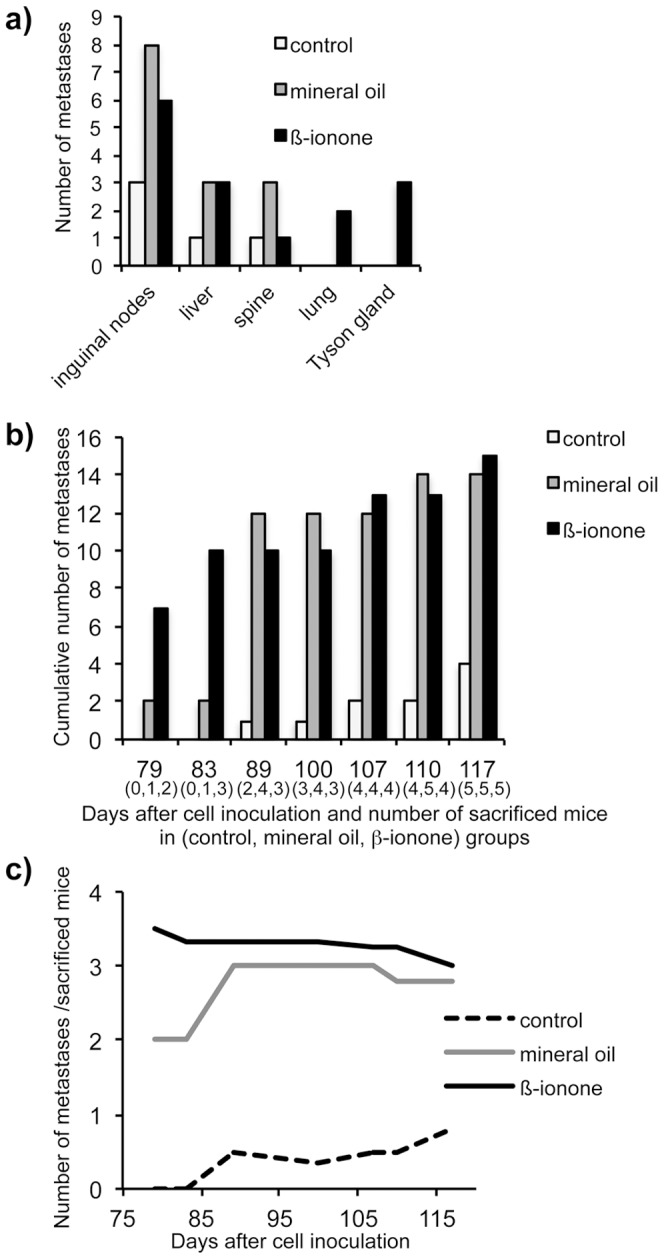
Metastases detection in immunodeficient mice inoculated with LNCaP cells. (**a**) The number of metastases observed in various tissues after mice sacrifice (white: untreated control mice, gray: mice treated with mineral oil, black: mice treated with 1 mM β-ionone in mineral oil). (**b**) Cumulative number of metastases, regardless of their location, as a function of the time elapsed between LNCaP cells inoculation and mice sacrifice (white: untreated control mice, gray: mice treated with mineral oil, black: mice treated with 1 mM β-ionone in mineral oil). Mice were sacrificed when tumor size exceeded 1,500 mm^3^. The number of sacrificed mice is indicated in brackets for each time point and each mice group. (**c**) Curves reporting the ratios of the number of metastases over the number of sacrificed mice as a function of the time (black dashed: untreated control mice, gray: mice treated with mineral oil, black: mice treated with 1 mM β-ionone in mineral oil).

Since *in vitro* enhancement of cell invasiveness by ORs activation suggests a possible role of (at least some) ORs in metastasis emergence *in vivo*, we inoculated LNCaP prostate tumor cells subcutaneously into immunodeficient NSG (NOD scid gamma) mice. Animals were either left untreated, or daily brushed on skin with PSGR agonist β-ionone diluted in mineral oil (an oily excipient needed to apply the lipophilic odorants over the mice skin), or with mineral oil alone as a control. Tumor size was measured and metastases were detected by *in vivo* imaging and by post-mortem immunohistochemistry using antibodies targeting PSGR or PSA (Prostate Specific Antigen) (examples of spine and lung metastases are displayed in Figure 4). PSGR expression was detected in primary tumors and in all metastases (see other examples in Figure S2), confirming that this receptor was present and possibly activated during our experiments. Without treatment, metastases emerged mainly in the inguinal nodes and occasionnally in spine and liver (Figure 4a). Metastases located in the inguinal nodes were well developed while those located in spine and liver were micrometastases. The number of metastases increased upon treatment with mineral oil and their localization was more diverse in the presence of β-ionone. Actually, metastases appeared in lungs and Tyson glands only for mice treated with β-ionone (3 out of 5 animals for Tyson glands and 2 out of 5 animals for lungs). Moreover, metastases located in Tyson glands were highly developed, with sizes approaching 1,000 mm^3^. In lungs, only micrometastases were detected, like in spine and liver. Since mice were not sacrificed at the same time, but depending on tumor size, we show in Figures 5b and 5c the evolution with time of the number of metastases according to the number of sacrificed mice and the average number of metastases per mouse at the time of sacrifice for each experimental group. We observed that mice treated with mineral oil or β-ionone had to be sacrificed earlier, due to faster tumor growth, and that they displayed a significant increase in metastases number compared to untreated mice. In parallel we also observed that tumors developed at 85% of the inoculated sites in untreated mice, while they were less numerous (50%) in mineral oil or β-ionone treated mice.

**Figure 6:**
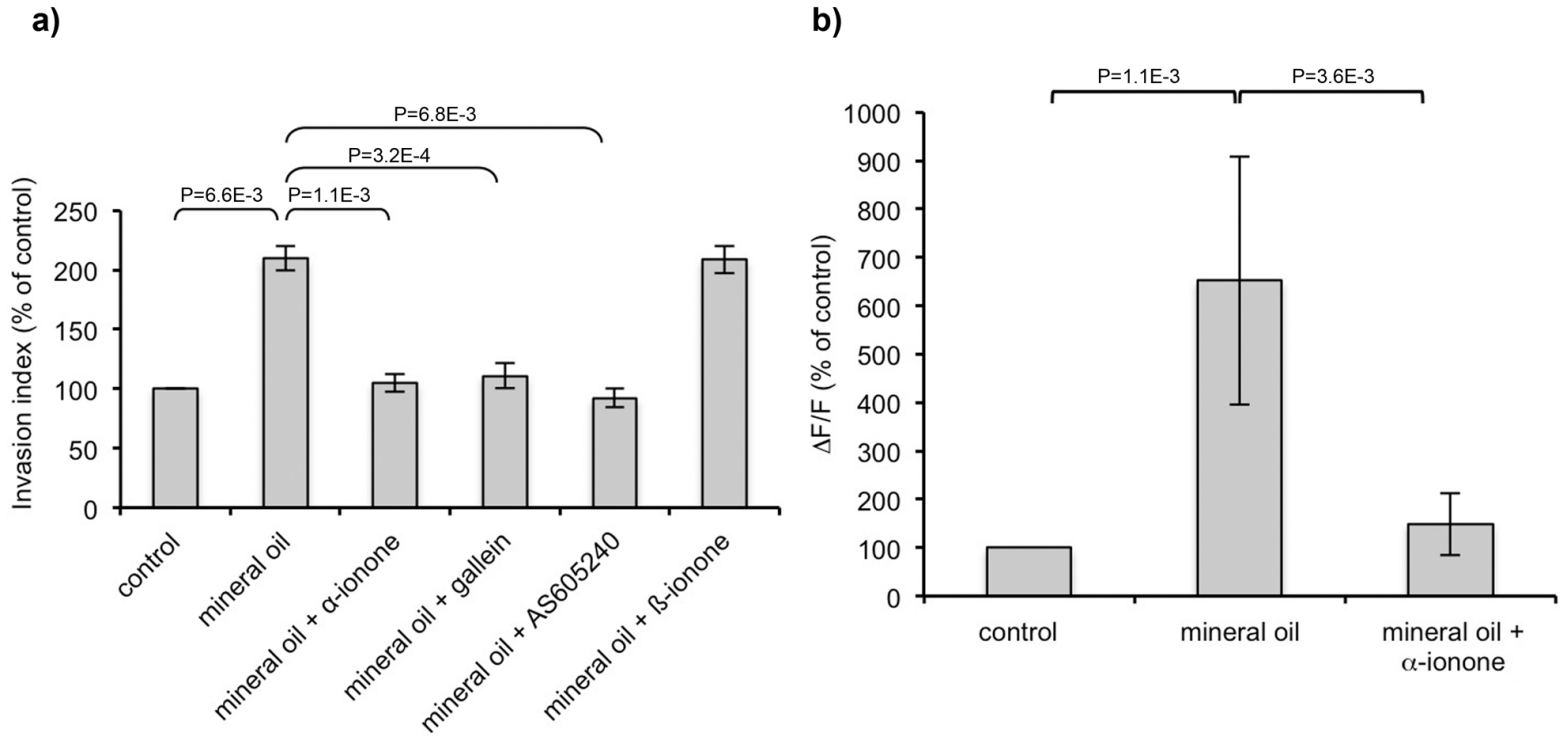
Mineral oil effect on LNCaP cells invasiveness and calcium response. (**a**) LNCaP cells were seeded onto collagen type I gels and were stimulated by mineral oil or mineral oil mixed with 10^−4^M β-ionone, 2.10^−4^M α-ionone, 10^−7^M AS605240 or 10^−5^M gallein. Mineral oil was first diluted 1/4 in DMSO (mineral oil was not totally solubilized at this dose, but this allowed us to expect a very large solubilization of the mineral oil components) and the solution was then diluted 1/1000 in the culture medium or collagen I gel. Invasive cells were counted 24 hours later. Results are presented as the invasion index relative to that of control cells (with only the DMSO). Bars indicate standard deviation. Standard deviation of the control was 3,67%. Results obtained with mineral oil alone and with mineral oil containing β-ionone are not significantly different. Statistics were performed using a two-tailed Student test (n = 3). (**b**) LNCaP cells were loaded with fluo-4 and stimulated with mineral oil or a mixture of mineral oil and 10^−4^M α-ionone. Calcium responses are expressed as the mean fluorescence variation ΔF/F relative to that of control, which corresponds to cells stimulated with buffer containing the same DMSO concentration than the odorants and mineral oil samples. Bars indicate standard deviation. Standard deviation of the control was 15,96%. Statistics were performed using a two-tailed Student test (n = 7).

The results with the mineral oil were intriguing. Since ORs can be activated by different molecules [2], [12], [18], we investigated whether mineral oil can stimulate the PSGR in *in vitro* cell invasion assays and calcium imaging experiments. Mineral oil increased both the invasiveness and the calcic response of LNCaP cells, and the PSGR antagonist α-ionone abrogated these effects (Figure 6 a,b). These results demonstrate that a mineral oil component stimulated LNCaP cells via the PSGR, promoting their invasiveness. Moreover, LNCaP cells invasiveness induced by mineral oil was inhibited by PI3Kγ or G_βγ_ subunits inhibitors (respectively AS605240 and gallein), demonstrating that PI3Kγ is involved in this process, as it is involved in cell invasiveness induced by β-ionone. However, even if mineral oil enhanced metastasis emergence, metastasis occurence was even more pronounced in the presence of β-ionone, resulting in the cells spreading to lungs and Tyson glands that occurred only in the presence of β-ionone. All these data converge to demonstrate that stimulation of an OR, the PSGR, can contribute to the dissemination of prostate tumor cells and in metastases generation.

## Discussion

In this study, we showed that contrary to OSNs, individual BON tumor cells (BON) co-express various ORs transcripts. Genes encoding the co-expressed ORs are not located in the same cluster nor on the same chromosomes, excluding a simultaneous expression controlled by a common promoter. OR co-expression by a same cell and differences in the set of expressed ORs between cells could ensure a wide detection of luminal odorants in the gastro-intestinal tract. Indeed normal and neoplastic EC cells were previously reported to detect odorants through OR activation, which participate in the control of their serotonin secretion [8], [9]. Deregulation of serotonin release can induce pathological disorders [29]-[32] and thus it could be expected that such disorders could result from changes in the levels and variety of the ORs expression. We also unexpectedly found that BON tumor cells express OR pseudogenes at the mRNA level. Some of them (OR7E38P and OR2A9P) and several functional receptors expressed by BON cells (OR7A17, OR7D2 and OR2A1) have been found in other tumors. Moreover, other ORs are overexpressed in EC tumor cells [15] and prostate tumor cells [12]. Thus, the expression of some OR genes and pseudogenes in BON cells, which originate from a pancreatic carcinoma metastasis, could be part of their tumor phenotype.

Since BON cells endogenously express ORs, we also demonstrated their ability to efficiently express transfected functional heterologous ORs with known odorant ligands. In particular, we found that the transcript of REEP1, a protein described to improve OR expression [20], was present in BON cells. These cells are thus a promising system for deorphanizing ORs, which still remains a challenge due to the well-known low and variable OR expression in heterologous cells. To date, even using HEK 293 cells engineered to improve OR expression, only 4% of 245 human ORs investigated were deorphanized [20], [33], [34]. Identifying OR ligands and studying OR pharmacology is of increasing interest given the high number of ORs (several hundreds in humans) and their involvement in various physiological functions, and possibly in physiopathological functions such as tumor progression.

Furthermore, we demonstrate, for the first time, that ORs play a role in tumor progression by promoting cell invasiveness and metatasis emergence. Taking advantage of the ability of BON cells to heterologously express ORs for which odorant ligands are known, we showed that odorant stimulation of heterologously expressed ORs enhanced BON cell invasiveness *in vitro*. We further assess this result using LNCaP prostate cancer cells, which overexpress an OR, the PSGR (also named OR51E2), described as a prostate tumor marker [11], [12]. Stimulation of LNCaP cells with the PSGR agonist β-ionone promoted their invasiveness, and this phenomenon was inhibited by α-ionone, a PSGR antagonist. Recently, ORs were reported to participate in early cytokinesis by exerting a regulatory role on actin cystoskeleton, and particularly in cancer cell lines [35]. This suggests that ORs could favor cell invasion by regulating actin cytoskeleton. Yet, the signaling pathway triggered by OR stimulation and inducing cell invasiveness remains to be explored. Our results provide a first evidence for a major role of PI3Kγ, which is also supported by the following data. The G_αolf_ protein subunit, activated by ORs in OSNs, was reported to promote invasion in human digestive and urogenital epithelial cells, and in particular in LNCaP cells, through PI3 kinase, Rho GTPases and PKC dependent pathways [36]. Yet, in our study, we found that PI3K is activated by βγ subunits of G proteins. Thus, in LNCaP cells, two PI3K dependent pathways originating from PSGR activation could coexist. Moreover, GPCRs activation of PI3Kγ is involved in transformed cell functions such as invasion and alteration of homotypic cell-cell adhesion [23], and a crosstalk between odorant signaling and PI3Kγ was reported in OSNs [24], [25]. All together, these data suggest that an interplay between ORs, G proteins and PI3Kγ in tumor cells might promote the cell invasiveness phenotype.

We also demonstrate *in vivo* that stimulation of some ORs expressed in tumor cells could facilitate cells dissemination and metastasis generation. Indeed, we stimulated xenografted LNCaP cells in NSG mice with a PSGR ligand. Without stimulation, mice developed tumors at 85% of the inoculation sites, but also some metastases in inguinal nodes, spine and liver, which is seldom reported in the literature concerning prostate cancer models using immunodeficient mice and LNCaP cells [37]–[39]. When treated with mineral oil alone or containing β-ionone, mice showed a significantly increased number of metastases, despite the small number of animals in the experiment. Noteworthy, only the β-ionone treated mice developed metastases in other tissues, namely in lungs and Tyson glands, and those in Tyson glands were particularly well developed. In addition, we showed that the mineral oil used as an excipient in our experiments induces LNCaP cell invasiveness by activating the PSGR and involving a PI3Kγ inhibitor pathway, like β-ionone. These results corroborate that metastases emergence enhancement observed in mineral oil treated mice can be due to PSGR activation, at least partly, since we cannot exclude the presence of other ORs in LNCaP cells and of their specific ligands in the mineral oil. It would be interesting to identify the mineral oil component activating the PSGR, but this appears difficult due to the complex and unavailable detailed composition of mineral oil. Moreover, while *in vitro* there was no additive effect of mineral oil and β-ionone in promoting cell invasion, *in vivo*, addition of β-ionone to mineral oil not only slightly boosted metastasis emergence in the same tissues but moreover induced metastasis spreading to additional tissues. Also, mice treated with β-ionone had to be sacrificed earlier due to faster tumor growth, and the number of metastases detected in those mice was sligthly higher. Thus, in the limit of our experimental conditions, where the real amount of mineral oil and β-ionone stimulating the LNCaP cells cannot be controlled, the presence of β-ionone appeared to further promote metastasis emergence. We cannot totally exclude that the observed effect on metastases emergence could also be partly due to an increased tumor growth rate in the presence of β-ionone. Indeed, β-ionone seemed to accelerate tumor growth, as suggested by the early sacrifice of some animals of the β-ionone treated group (actually, all the animals were sacrificed when their largest lesion – the inoculated tumor or a metastasis – reached 1500 mm^3^). However, the treated animals sacrificed earlier presented more metastases than untreated animals sacrificed later, whereas it could be expected the contrary since the number of metastases usually increase with age. Moreover, there were no significant differences in tumor size between mice groups at sacrifice time. So, the observed differences in the number and localization of the metastases cannot be attributed just to differences in tumor growth. We can also notice that the faster tumor growth observed *in vivo* in the presence of β-ionone is not in agreement with the results of Neuhaus et al. [12] demonstrating a reduced *in vitro* proliferation of LNCaP cells in the presence of β-ionone. Finally, we observed that tumor engraftement was less important in treated mice than in untreated mice. This could be due to a greater migration of the cells stimulated with mineral oil or β-ionone from the inoculation site, detrimental to the local tumor development.

In humans, the PSGR can be activated by endogenous ligands such as steroid hormones [12]. Nevertheless, we performed this study in an androgen-depleted context (that is in castrated male mice) and therefore our findings are of major relevance concerning the androgen-independent progression of prostate cancer, which is still poorly understood. Since mineral oil and β-ionone are present in various products of our close environment, namely cosmetics, food and beverages, it is conceivable that these exogenous molecules could be found in the body and our findings might help defining prostate cancer prevention measures. An important prospect of future studies would be also to identify PSGR odorant antagonists (other than α-ionone which is irritant and harmful, side effects that would limit its use) as potential new anti-cancer agents probably possessing low side effects since the PSGR is not widely expressed in normal tissues.

Finally this study should be extended to other cancer types. Indeed, not only a genomics approach has associated the olfactory transduction pathway with an increased pancreas cancer risk [40] but moreover overexpression of 34 ORs genes has been reported in patients bearing breast tumors caused by CHEK2 1100delC-mutation [41]. Hence, it appears of great importance to continue addressing the role of ORs in tumor progression, in hormono-dependent tumors as well as in non hormono-dependent ones.

## Supporting Information

Figure S1 **ORs expression in BON cells.** (**a**) Example of nested PCR performed on RT products obtained from a single BON cell from clone 14, using two pairs of primers specifically targeting OR10Q1 or OR4F16. Each amplification product was was about 400 bp long and was verified by sequencing. Lane 1: 2-Log DNA Ladder (BioLabs), lane 2: OR4F16 amplification, lane 3: OR10Q1 amplification, lanes 4 and 5: controls without cell with each pair of primers. (**b**) Heterologous expression of ORs by BON cells. BON cells were transiently transfected to express OR1G1 or OR17-40 receptors tagged by the cmyc epitope at their extracellular N-terminal end. 72h later, non-permeabilised cells, labeled with an anti-cmyc-Cy3 antibody, were observed by immunofluorescence microscopy. Mock-transfected cells are shown as a negative control. (**c**) REEP1 transcripts in BON cells. RT-PCRs were performed using total RNA from BON cells and primers targeting human RTP1, RTP2 or REEP1 cDNAs. Expected PCR products lengths were 686 bp for RTP1, 564 bp for RTP2 and 524 bp for REEP1. Negative controls were obtained with the same primers on RNAs treated without reverse transcriptase. Only REEP1 cDNA was amplified at the expected length (boxed in white).
(TIF)

Figure S2 **PSGR expression in various tissues of mice inoculated with LNCaP cells.** PSGR expression in primary tumors, inguinal nodes, Tyson glands and livers was detected using an anti-PSGR antibody (LS-A6332, Cliniscience) and negative controls were performed using rabbit serum.
(TIF)

Table S1 **Blind search of ORs expressed by subclones of BON cells, using nested RT-PCR with degenerate primers.** Alternative ORs denominations are given in brackets. « P » indicates pseudogenes.
(DOCX)
