# Supplementary figures and images for: Promotion of Cancer Cell Invasiveness and Metastasis Emergence Caused by Olfactory Receptor Stimulation
Source: PLoS One. 2014 Jan 8;9(1):e85110. doi: 10.1371/journal.pone.0085110 (PMC3885679; doi:10.1371/journal.pone.0085110)

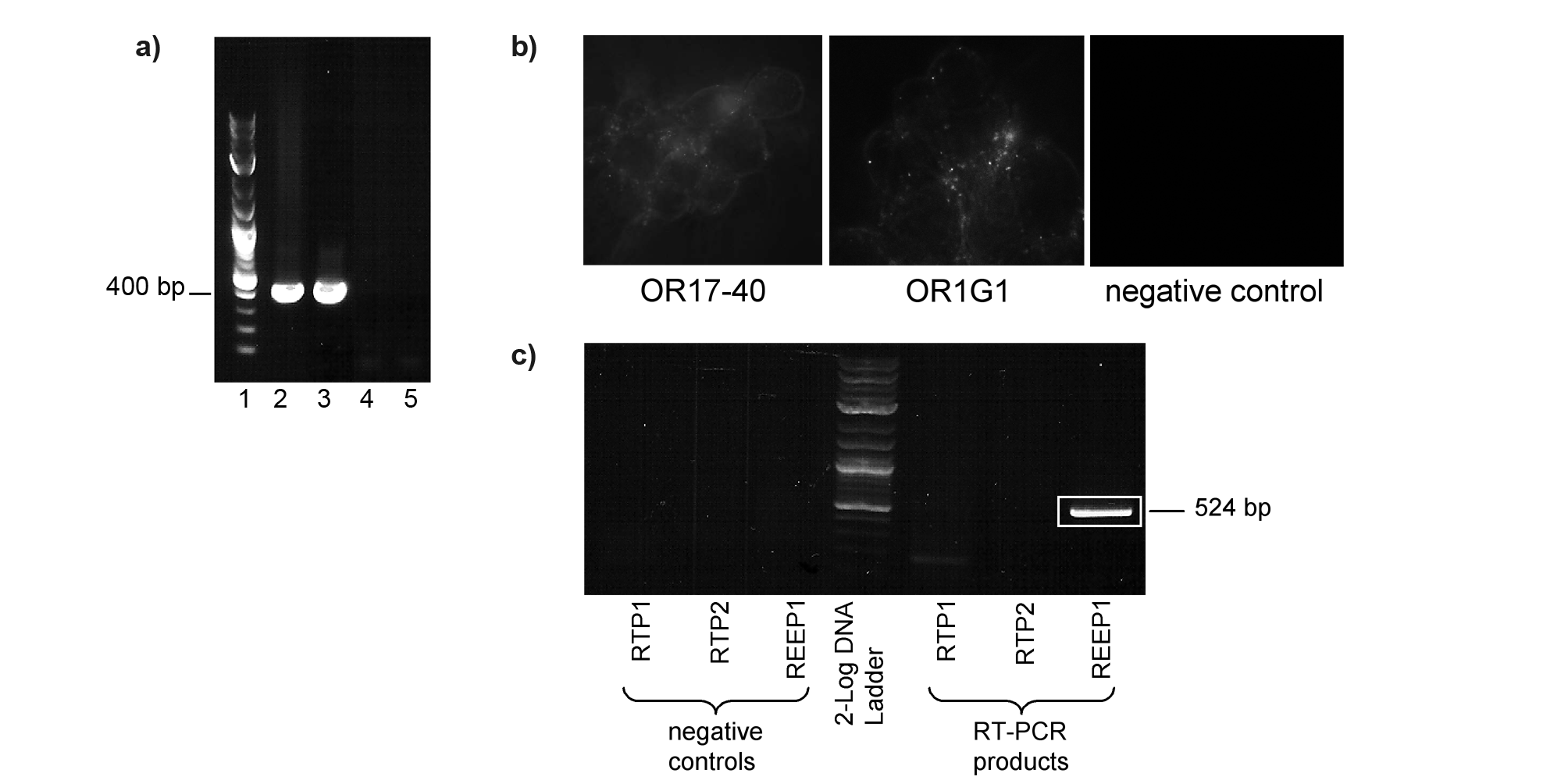

Supplement: Figure S1 — ORs expression in BON cells. (a) Example of nested PCR performed on RT products obtained from a single BON cell from clone 14, using two pairs of primers specifically targeting OR10Q1 or OR4F16. Each amplification product was was about 400 bp long and was verified by sequencing. Lane 1: 2-Log DNA Ladder (BioLabs), lane 2: OR4F16 amplification, lane 3: OR10Q1 amplification, lanes 4 and 5: controls without cell with each pair of primers. (b) Heterologous expression of ORs by BON cells. BON cells were transiently transfected to express OR1G1 or OR17-40 receptors tagged by the cmyc epitope at their extracellular N-terminal end. 72h later, non-permeabilised cells, labeled with an anti-cmyc-Cy3 antibody, were observed by immunofluorescence microscopy. Mock-transfected cells are shown as a negative control. (c) REEP1 transcripts in BON cells. RT-PCRs were performed using total RNA from BON cells and primers targeting human RTP1, RTP2 or REEP1 cDNAs. Expected PCR products lengths were 686 bp for RTP1, 564 bp for RTP2 and 524 bp for REEP1. Negative controls were obtained with the same primers on RNAs treated without reverse transcriptase. Only REEP1 cDNA was amplified at the expected length (boxed in white). (TIF) [file pone.0085110.s001.tif]

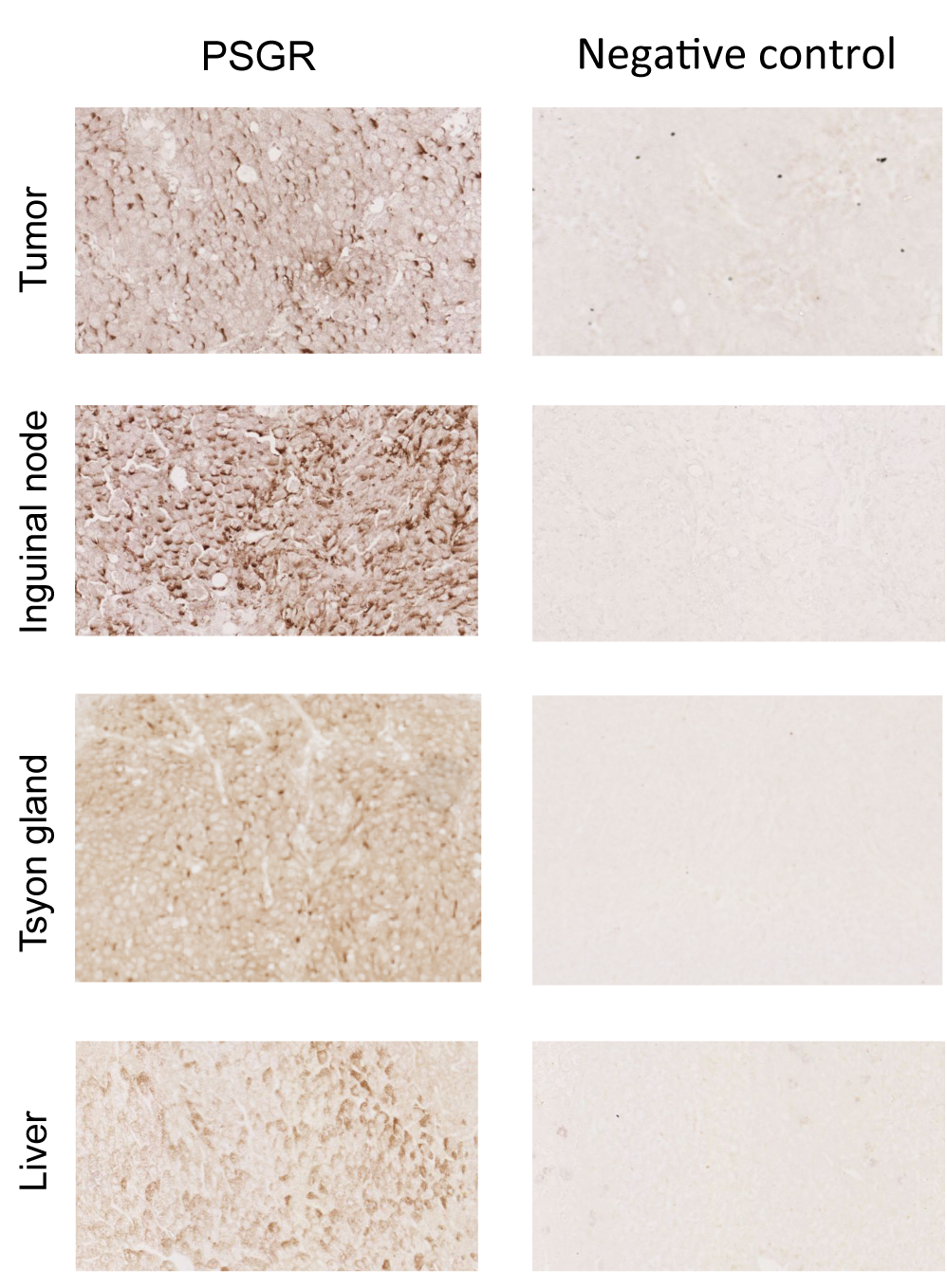

Supplement: Figure S2 — PSGR expression in various tissues of mice inoculated with LNCaP cells. PSGR expression in primary tumors, inguinal nodes, Tyson glands and livers was detected using an anti-PSGR antibody (LS-A6332, Cliniscience) and negative controls were performed using rabbit serum. (TIF) [file pone.0085110.s002.tif]
