# Supplementary material for: Promotion of Cancer Cell Invasiveness and Metastasis Emergence Caused by Olfactory Receptor Stimulation
Source: PLoS One. 2014 Jan 8;9(1):e85110. doi: 10.1371/journal.pone.0085110 (PMC3885679; doi:10.1371/journal.pone.0085110)
Supplement: Table S1 — Blind search of ORs expressed by subclones of BON cells, using nested RT-PCR with degenerate primers. Alternative ORs denominations are given in brackets. « P » indicates pseudogenes. (DOCX) [file pone.0085110.s003.docx]

**Supplementary Table S1.** **Blind search of ORs expressed by subclones of BON cells, using nested RT-PCR with degenerate primers.**

Alternative ORs denominations are given in brackets. « P » indicates pseudogenes.

| **Clone**  **number** | **Identified ORs** |
| --- | --- |
| **4** | OR4F16 (= OR4F3, OR4F29, OR4F21) |
| **9** | OR7E91P, OR7E99P |
| **10** | OR13H1, OR7E99P |
| **14** | OR7A17, OR10Q1, OR7D2 |
| **19** | OR1F1, OR13A1, OR7E38P, OR2A9P (=OR2A20P) |
| **23** | OR2A1 (=OR2A42), OR6V1, OR1F1, OR13A1, OR2A9P (=OR2A20P) |
